# Comparative Analysis of Meat Quality in Minxinan Black Rabbit and Hyla Rabbit Using Integrated Transcriptomics and Proteomics

**DOI:** 10.3390/ani15243616

**Published:** 2025-12-16

**Authors:** Weiwei Mi, Lei Sang, Yajia Zhang, Gongyan Liu, Liping Yang, Haitao Sun, Haihua Zhang, Guanhua Fu, Chengfang Gao, Liya Bai

**Affiliations:** 1 Key Laboratory of Livestock and Poultry Multi-Omics of MARA, Institute of Animal Science and Veterinary Medicine, Shandong Academy of Agricultural Sciences, Jinan 250100, China; miweiv@163.com (W.M.); 19713242684@163.com (Y.Z.); gongyanliu@foxmail.com (G.L.); yanglp682@163.com (L.Y.); wwww8888@163.com (H.S.); 2 College of Life Science and Food Engineering, Hebei University of Engineering, Handan 056038, China; guanhua1220@163.com; 3 Fujian Key Laboratory of Animal Genetics and Breeding, Institute of Animal Husbandry and Veterinary Medicine, Fujian Academy of Agricultural Sciences, Fuzhou 350013, China; sanglei1981@163.com (L.S.); gaochengfang602@163.com (C.G.); 4 Hebei Key Laboratory of Specialty Animal Germplasm Resources Exploration and Innovation, College of Animal Science and Technology, Hebei Normal University of Science and Technology, Qinhuangdao 066000, China; zhh83@126.com

**Keywords:** Minxinan black rabbit, meat quality, proteomics, glutathione metabolism, fatty acid biosynthesis

## Abstract

The Minxinan black rabbit (MBR) is a valuable genetic resource in China. In this study, we compared meat quality between indigenous MBR and introduced Hyla rabbit (CIR) using multi-omics and phenotypic analyses. We identified 1175 differentially expressed genes (DEGs) and 327 differentially expressed proteins (DEPs) with 32 overlapping. Antioxidant-related pathways (glutathione metabolism, peroxidase activity, and HIF-1) were enriched, with upregulated superoxide dismutase 1 (SOD1) and gamma (γ)-glutamylcyclotranserase (GGCT) (*p* < 0.05), enhancing the antioxidant capacity in MBR. Meanwhile, key proteins (GSTM3, ENSOCUG00000024443, and ENSOCUG00000009681) in the glutathione and cytochrome P450 pathways may regulate meat color by reducing oxidative stress. Phenotypic analysis confirmed that the MBRs had increased redness (a*), yellowness (b*), melanin, and myoglobin (*p* < 0.05). Additionally, proteins involved in fatty acid metabolism (ACSL6), purine metabolism (NME2 and NME4), tight junctions (MYL10), and amino acid biosynthesis (ENO1) are involved in regulating fatty acid content, which can influence meat flavor quality. Notably, MBR meat exhibited significantly increased levels of unsaturated fatty acids (UFAs) and glycine (*p* < 0.05), which regulate meat flavor. MBR demonstrated superior antioxidant capacity, color, and flavor, thus providing a scientific foundation for optimizing conservation strategies.

## Simple Summary

Rabbit meat is considered a superior choice among livestock and poultry meats due to its high protein, high lysine, and high digestibility, as well as its low fat, low cholesterol, and low calorie content. Genetic factors underlying the distinctive meat flavor and other traits of Minxinan black rabbit (MBR), a Chinese indigenous rabbit, remain poorly characterized. The genetic basis for the superior meat quality of the indigenous MBR was investigated by comparing it with the introduced Hyla rabbit (CIR) using multi-omics and phenotypic analyses. MBR demonstrated better antioxidant capacity, flavor, fatty acid profile, and meat color (elevated redness (a*), yellowness (b*), melanin and myoglobin), underscoring its value as a premium food and breeding resource. These traits are linked to key pathways, particularly glutathione metabolism, playing a pivotal role in regulating meat quality, concurrently modulating antioxidant activity and meat color, where novel genes (e.g., *ENSOCUG00000024443*) and proteins (e.g., GGCT, SOD1) were identified. Enhanced flavor was associated with altered fatty acid and purine metabolism, mediated by proteins such as ACSL6 and NME2/NME4. We believe that this study provides a theoretical foundation for supporting MBR meat consumption and selecting MBR as a breeding material for future rabbit breeds, offering consumers a more comprehensive reference when selecting livestock and poultry meats.

## 1. Introduction

Rabbit meat provides an ideal source of high-quality protein, essential fatty acids, vitamins, and minerals and is widely consumed in certain regions worldwide [1,2]. China holds a dominant position in global rabbit production, contributing to more than half of the world’s total output [3]. Rabbit breeding is a distinctive livestock industry in China; however, the sector relies predominantly on foreign-developed breeds [4]. Currently, the Hyla rabbit (CIR), an artificially bred white fur variety that was introduced from abroad, commands the dominant share of China’s domestic meat rabbit market [5]. Severe protection and underutilization of local genetic resources (such as Minxinan black rabbit (MBR), Fujian yellow rabbit, Laiwu black rabbit, etc.) have constrained the domestic breeding capacity of rabbit meat breeds.

The MBR, a superior local genetic resource predominantly found in Longyan City, Fujian Province, southern China, was officially recognized by the National Commission for Livestock and Poultry Genetic Resources in 2010 and subsequently included in the China Livestock and Poultry Genetic Resources list in 2021 as a local rabbit breed [6]. This premium indigenous Chinese meat rabbit breed demonstrates broad adaptability, strong disease resistance, tolerance to coarse feed, high reproductive performance, and superior meat quality [5,7]. MBR meat contains approximately double the amino acid, protein, and melanin contents compared to those of conventional rabbits [8]. Sang et al. [9] demonstrated that 90-day-old MBR and Fujian white rabbits exhibited higher intramuscular fat content than that of New Zealand rabbits, and the MBR showed a more reddish meat color than that of Fujian white and New Zealand rabbits. Zhou et al. [5] showed that the myoglobin content in MBR meat was significantly higher than that in CIR meat. However, challenges persist in conserving and developing local livestock genetic resources, including population decline, inadequate exploitation of advantageous traits (meat quality and stress resistance), ambiguous breeding objectives, and slow selection progress. Our research group introduced the MBR to Shandong Province through the “Southern Breeds Northern Multiplication” initiative under Shandong Province’s Improved Breeds Project. The optimal slaughter age for this breed was 110 to 130 days [10]. Preliminary assessments of the growth characteristics and meat quality indicators revealed promising characteristics [11,12]. However, genetic determinants underlying its distinctive meat flavor and other traits remain incompletely characterized.

The assembly and publication of the reference genome for domestic rabbits by Carneiro et al. [13] have provided a foundation for comprehensive genome-wide studies of rabbit genetic traits. The integration of multiple omics approaches, such as genetic, transcriptional, and protein-level analyses, enables a more systematic and comprehensive exploration into the genetic mechanisms underlying biological traits. Wang et al. [14] observed that CIR exhibited higher intramuscular fat content than that of Champagne and Tianfu Black rabbits. Previous studies on the genetic diversity and population structure of domestic and imported rabbit breeds have primarily employed techniques such as RAD-seq [4]. Multi-omics integrated analysis facilitates a comprehensive assessment of gene expression levels and can reveal novel findings that cannot be demonstrated through conventional single-omics approaches. Huang et al. [15] demonstrated the regulatory mechanisms of hair fiber diameter in Angora rabbits using integrated transcriptomic and proteomic analyses. Kuang et al. [16] conducted a multi-omics analysis of the longissimus thoracis et lumborum (LTL) of Shuxing No.1 rabbits and CIR, revealing that the tryptophan metabolism pathway, associated with meat quality and flavor, was enriched with numerous differentially expressed genes (DEGs), differentially expressed proteins (DEPs), differentially accumulated metabolites (DAMs), and melatonin, which elucidated the potential mechanisms underlying meat quality and flavor.

In the present study, we employed a combined analysis of transcriptomics and proteomics to compare the differences in meat quality and oxidative stability between the local MBR breed and introduced CIR. Physical meat traits and relevant meat quality indicators, such as amino acid and fatty acid compositions, were measured and analyzed. This study provides a comprehensive analysis of the differences in meat quality between the local MBR and introduced CIR breeds from both genetic and phenotypic perspectives. These findings elucidate the superior genetic traits of MBR, thereby providing theoretical guidance for the utilization and promotion of this breed as well as establishing a foundation for the selection and breeding of new rabbit breeds.

## 2. Materials and Methods

### 2.1. Animals and Sample Collection

Rabbits of the MBR and CIR breeds were chosen to be divided into two groups based on breeds. The MBR group and CIR group, each including 20 rabbits, which were all originating from maternal rabbits who were not litter or half-siblings, weaned at 35 days of age with similar body weights. Each rabbit was individually housed (60 × 41 × 35 cm), all rabbits were housed in a closed, ventilated room (temperature: 20–25 °C, relative humidity: 50–60%) at Huifu Agriculture and Animal Husbandry Co., Ltd., which was located in Chiping District, Liaocheng City, Shandong Province, China, under identical management conditions, with free access to feed and water. The basic diet composition and nutritional level of MBR and CIR (Supplementary Table S1), with reference to Zhou et al. [5]. After 80 days, the rabbits were subjected to a 24–h fast without food or water. Ten rabbits with similar weight and good health were randomly slaughtered per group, and the LTLs were collected to determine the relevant meat quality traits. The LTLs of 3 rabbits were randomly selected from each group and stored at –80 °C for subsequent RNA and protein extraction.

### 2.2. Transcriptional Extraction and Data Processing

Total RNA was extracted from the LTLs of the three MBR and CIR using an RNA extraction kit (Tiangen Biotech, Beijing, China). The purity and integrity of the RNA were determined using agarose gel electrophoresis, ultraviolet spectrophotometry, and biological analysis. Using the structural characteristics of most mRNAs in eukaryotes with poly(A) tails, mRNAs with poly(A) tails were enriched using oligo (dT) magnetic beads, the mRNAs were randomly interrupted, and the library was constructed according to the NEB common library construction method. The library was subjected to Illumina sequencing. The sequencing fragments were converted into sequence data (reads) in the FASTQ file format by Base Calling. The reference genome was Ensembl_oryctolagus_cuniculus_orycun2_0_gca_000003625_1. We used HISAT2 v2.0.5 [17] to construct the index of the reference genome and align the paired-end clean reads with the reference genome.

### 2.3. Total Protein Extraction and Data Processing

Proteins were extracted using a protein extraction kit (Beyotime Biotechnology, Shanghai, China) according to the manufacturer’s instructions. A Bradford protein quantification kit (Bio-Rad Laboratories, Hercules, CA, USA) was used to determine the protein concentration and draw the labeling curve, and the protein concentration of the sample to be tested was calculated. The library was prepared by the Novogene Co., Ltd. (Beijing, China). The general procedure involved determining the total protein quality, trypsin digestion, Tandem Mass Tag (TMT) labeling after desalination, fraction separation, and mass spectrometry.

Based on the Raw file obtained by mass spectrometry detection, the corresponding database was searched, and proteins were identified based on the results of the database 466682-Oryctolagus_cuniculus.orycun2.59.pep.all.fasta (23,910 sequences). Simultaneously, the mass tolerance distribution of peptides, proteins, and parent ions was analyzed to evaluate the quality of the mass spectrometry detection data. The identified proteins were annotated using common functional databases and quantitative analysis of proteins, including overall difference analysis of identified proteins, screening of differential proteins, and cluster analysis of expression patterns.

### 2.4. Combined Analysis of Transcriptome and Proteome

The transcriptome and proteome data were associated with the same reference gene data. When the transcriptome and proteome detected the same gene expression simultaneously, a relationship existed between the mRNA and protein levels of the gene. Using the GO and KEGG functional enrichment of the proteome as a reference, the same GO and KEGG entries in the transcriptome were screened, and the biological signaling pathways involved were screened according to the order of *p*-values from smallest to largest.

### 2.5. Construction of Protein Interaction Network

The STRING database (https://string-db.org/, accessed on 10 January 2025) was used to establish an interaction network diagram between the candidate proteins, and the key interacting proteins were screened. The interaction proteins were imported into Cytoscape 3.10.1 software [18] for visual editing.

### 2.6. Determination of Meat Quality

#### 2.6.1. Meat Color

Objective colors, including a* (redness), b* (yellowness), and L* (lightness), were measured 45 min after slaughter using a colorimeter (NR20XE, 3nh, Shenzhen, China) along with reflectance spectroscopy of the fresh cross-section of the LTLs muscle.

#### 2.6.2. Nutritional Components

According to the method specified in GB 5009.3-2016 [19], muscle samples should be labeled, dried until they reach a constant weight, and then the dry matter content should be determined. The crude protein content was determined using the Kjeldahl method in accordance with GB 5009.5-2016 [20]. The crude fat content of the muscle was measured using the Soxhlet extraction method in accordance with GB 5009.6-2016 [21]. The glycine content of the muscle was analyzed using an LA8080 amino acid analyzer (Hitachi High-Tech Corporation, Tokyo, Japan), based on GB 5009.124-2016 [22].

#### 2.6.3. Fatty Acid Composition and Content

Fatty acid analysis was conducted by hydrolyzing muscle samples, fat extraction, saponification, and fatty acid methyl ester (FAME) derivatization, followed by Gas chromatography–mass spectrometry (GC–MS) analysis. An Agilent 7890A gas chromatography system (Agilent Technologies Inc., Santa Clara, CA, USA) was used for GC-MS. Separation was achieved on an HP-88 Chromatographic column (100 m × 0.25 mm × 0.20 μm) with a programmed temperature gradient: 100 °C (13 min hold), ramped to 180 °C at 10 °C/min (6 min hold), then to 192 °C at 1 °C/min (9 min hold), and finally to 230 °C at 3 °C/min (10 min hold). The injection port was set at 240 °C in split mode (split ratio 0.8). Detection was performed by FID at 280 °C, using nitrogen as the carrier gas at 1.3 mL/min.

#### 2.6.4. Enzyme-Linked Immunosorbent Assay (ELISA)

The melanin, superoxide dismutase 1 (SOD1), and gamma (γ)-glutamylcyclotranserase (GGCT) levels in the LTL were quantified using ELISA kits (Kete Biological, Taizhou, Jiangsu, China). Absorbance was measured at 450 nm, and standard curves were plotted to calculate the actual sample concentrations.

#### 2.6.5. Myoglobin Content

The sample (0.2 g) was homogenized in 1 mL of 0.04 mol/L (pH = 6.8) phosphate solution. The sample was placed on ice for 1 h, centrifuged at 3500× *g* for 20 min at 4 °C, and the supernatant was placed in a 96-well plate. Myoglobin concentration (nmol/mL) was calculated from absorbance measurements at 525, 545, 565, and 572 nm using the formula [5]:

Myoglobin = (−0.166 A_572 nm_ + 0.086 A_565 nm_ + 0.088 A_545nm_ + 0.099 A_525 nm_) × 1000

### 2.7. Statistical Analysis

Differential expression analysis between the two breeds was performed using DESeq2 1.16.1 software [23]. The resulting *p*-values were adjusted using Benjamini and Hochberg’s approach to control for the false discovery rate. Genes with |log2FoldChange| > 1.0 and an adjusted *p*-value < 0.05 were assigned as differentially expressed. The resulting RAW files were analyzed using Proteome Discoverer 2.2 software [24]. Proteins were considered significantly upregulated if *p* ≤ 0.05 and fold change (FC) ≥ 1.2, and significantly downregulated if *p* ≤ 0.05 and FC ≤ 0.83. The Pearson correlation coefficient of DEGs and DEPs was calculated, and proteins (genes) and correlations with significant differences between the two groups were obtained. Cluster Profiler 3.4.4 software [25] was used to perform KEGG (http://www.genome.jp/kegg/, accessed on 1 January 2025) or GO (http://www.geneontology.org/, accessed on 2 January 2025) enrichment analyses of the DEGs or DEPs. The functional significance enrichment threshold was set at *p* < 0.05. The enriched pathways associated with the target traits were presented in the form of bubble plots.

The meat quality data were tested for normality (Shapiro–Wilk test) and homogeneity of variance (Levene’s test) using SPSS 26.0 software (IBM Corp., Armonk, NY, USA), confirming that the data satisfied both conditions (*p* > 0.05). Therefore, an independent samples *t*-test was performed for statistical analysis, with results expressed as mean ± standard deviation. Statistical significance was set at *p* < 0.05. Graphs were generated using GraphPad Prism 9.5 (GraphPad Software, Inc., La Jolla, CA, USA).

## 3. Results

### 3.1. Transcriptome, Proteome, and Two-Omics Combined Expression Regulation Analysis of MBR and CIR Meat

**Figure 1:**
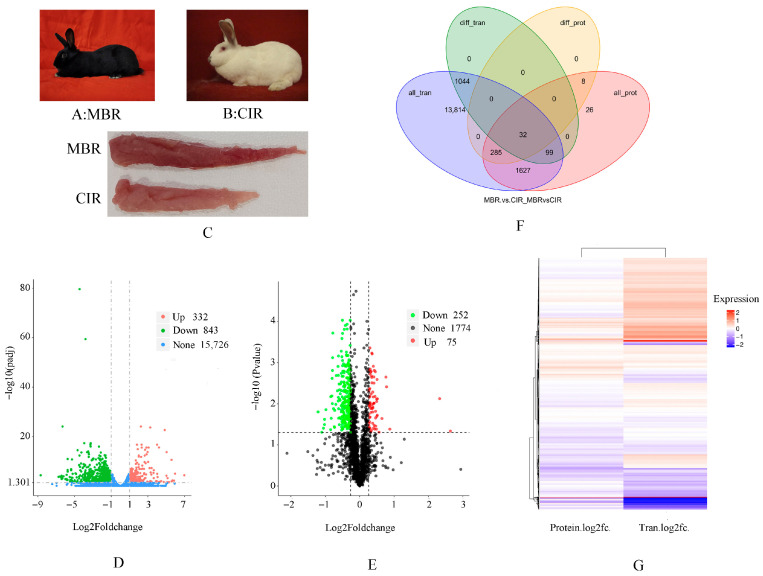
Differentially expressed genes (DEGs) and differentially expressed proteins (DEPs) between Minxinan black rabbit (MBR) and Hyla rabbit (CIR). (**A**) Minxinan black rabbit. (**B**) Hyla rabbit. (**C**) The longissimus thoracis et lumborum (LTL) muscle of MBR and CIR. (**D**) The volcano plot of DEGs for MBR vs. CIR: red represents upregulation, green represents downregulation, and blue/black represents no significant difference. (**E**) The volcano plot of DEPs for MBR vs. CIR: red represents upregulation, green represents downregulation, and black represents no significant difference. (**F**) The Venn diagram of DEGs and DEPs for MBR vs. CIR. (**G**) Heatmap analysis of DEGs and DEPs for MBR vs. CIR. The Tran represents transcriptomics, and the Protein represents proteomics. Same below.

At the mRNA level, compared to those of CIR, MBR exhibited 332 upregulated genes, 843 downregulated genes, and a total of 1175 DEGs were enriched (*p* ≤ 0.05, |log2 Foldchange| ≥ 1) (Figure 1D, Supplementary Table S2). Compared to those of CIR, MBR exhibited 75 upregulated proteins (*p* ≤ 0.05, FC ≥ 1.2), and 252 downregulated proteins (*p* ≤ 0.05, FC ≤ 0.87). A total of 327 proteins were differentially expressed between the MBR and CIR groups (Figure 1E, Supplementary Table S2).

RNA-seq and TMT quantitative proteomics were combined and analyzed. Based on the above conditions, 32 significantly differentially co-expressed proteins (genes) were observed at the proteome and transcriptome of MBR and CIR (Figure 1F). Correlation analysis was performed on different multiples of proteins (genes) identified together in the two omics. The heat map (Figure 1G) shows that the upregulated and downregulated expression patterns of most genes and proteins were similar.

### 3.2. KEGG Functional Enrichment Analysis

**Figure 2:**
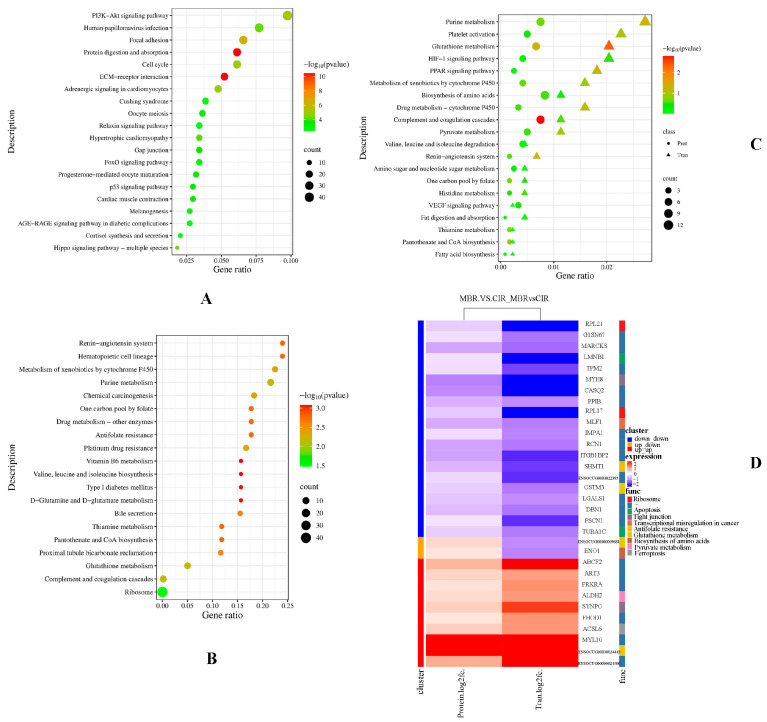
KEGG enrichment analysis of the DEGs and DEPs between the MBR and CIR. (**A**) Bubble chart of DEGs in KEGG. (**B**) Bubble chart of DEPs in KEGG. (**C**) KEGG enrichment bubble chart of DEGs and DEPs. (**D**) Heatmap analysis of DEGs and DEPs for MBR vs. CIR in KEGG. The *x*-axis represents the Gene ratio. The *y*-axis represents the KEGG. The size of the bubble/triangles represents the number of genes annotated to a KEGG, and the color represents the enrichment *p* value, where the darker the color, the smaller the *p* value. Same below.

Cluster Profiler 3.4.4 software [25] was used to analyze KEGG pathway enrichment of the transcriptome and proteome of the two rabbit breeds, MBR and CIR. The top 20 pathways were significantly enriched in the transcriptome (*p* < 0.05, Figure 2A, Supplementary Table S3). The main significantly enriched pathways were as follows: ECM-receptor interaction, focal adhesion, PI3K-Akt signaling pathway, Hippo signaling pathway, progesterone-mediated oocyte maturation, oocyte meiosis, and melanogenesis. In the proteome, two significantly enriched pathways (*p* < 0.05) were the ribosome and complement and coagulation cascades. When *p* > 0.05, the main enrichment pathways were as follows: glutathione metabolism; valine, leucine, and isoleucine biosynthesis; vitamin B6 metabolism; one-carbon pool by folate; antifolate resistance; metabolism of xenobiotics by cytochrome P450; and renin-angiotensin system (Figure 2B).

A total of 55 KEGG pathways were enriched using a combination of the two-omics. We selected 20 traits related to those of target to draw a bubble diagram (Figure 2C, Supplementary Table S3). Specifically, the pathways enriched, which may be related to oxidative stability, were complement and coagulation cascades, glutathione metabolism, renin-angiotensin system, platelet activation, and HIF-1 signaling pathway. The possible pathways related to meat color were metabolism of xenobiotics by cytochrome P450 and drug metabolism-cytochrome P450. Purine metabolism, fatty acid biosynthesis, fat digestion and absorption, amino sugar and nucleotide sugar metabolism may be related to meat flavor. Thiamine metabolism, one-carbon pool by folate, biosynthesis of amino acids, valine, leucine and isoleucine degradation, and histidine metabolism pathways affect amino acid and vitamin metabolism in meat. Notably, broad-spectrum pathways, including the PPAR and VEGF signaling pathways, were identified.

A heat map was drawn based on the expression levels of the 32 differentially expressed proteins (genes) and the enriched KEGG pathways (Figure 2D, Supplementary Table S3). The main enrichments were for glutathione metabolism (*GSTM3*, *ENSOCUG00000024443,* and *ENSOCUG00000009681*), tight junctions (*SYNPO* and *MYH8*), ferroptosis (*ACSL6*), biosynthesis of amino acids (*ENO1*), apoptosis (*LMNB1*), ribosomes (*RPL21* and *RPL17*), and antifolate resistance (*SHMT1*).

### 3.3. GO Functional Enrichment Analysis

**Figure 3:**
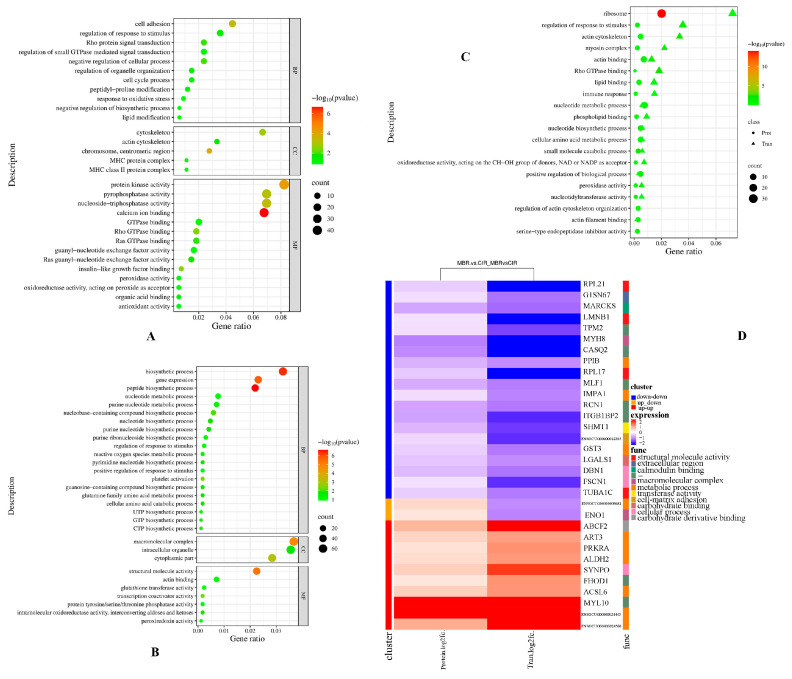
GO enrichment analysis of the DEGs and DEPs between the MBR and CIR. (**A**) Bubble chart of DEGs in GO. (**B**) Bubble chart of DEPs in GO. (**C**) GO enrichment bubble chart of DEGs and DEPs. (**D**) Heatmap analysis of DEGs and DEPs for MBR vs. CIR in GO.

Cluster Profiler 3.4.4 software [25] was used to analyze the GO functional enrichment of the transcriptome and proteome of the two rabbit breeds, MBR and CIR (Supplementary Table S4). Thirty GO terms related to the traits of target were selected to construct the bubble diagram. Seventeen GO terms were significantly enriched within the biological process (BP), cell composition (CC), and molecular function (MF) GO categories (Figure 3A; *p* < 0.05). The enriched terms were functionally clustered into: (1) molecular binding and catalytic activities (calcium ion binding, nucleoside-triphosphatase activity, Rho GTPase binding, Ras GTPase binding, insulin-like growth factor binding, protein kinase activity, pyrophosphatase activity, and Ras guanine nucleotide exchange factor activity), (2) cellular architecture and dynamics (cytoskeleton organization, actin cytoskeleton assembly, chromosome centromeric region, MHC protein complex, negative regulation of cellular processes, regulation of response to stimulus, and regulation of organelle organization), and (3) redox homeostasis and metabolic processes (peroxidase activity, antioxidant activity, response to oxidative stress, oxidoreductase activity, acting on peroxide as acceptor, and lipid modification). Proteomic analysis revealed 17 significantly enriched GO terms (Figure 3B; *p* < 0.05), which were functionally classified into three major clusters: (1) biosynthetic processes (peptide biosynthetic process, nucleobase-containing compound biosynthesis, nucleotide biosynthesis, pyrimidine nucleotide biosynthesis, purine nucleotide biosynthesis, and guanosine-containing compound biosynthesis), (2) structural and compartmental organization (macromolecular complex assembly, structural molecule activity, cytoplasmic part, and intracellular organelle), and (3) metabolic regulation (glutamine family amino acid metabolic process, cellular amino acid catabolic process, intramolecular oxidoreductase activity, aldose-ketose interconversion, platelet activation, and transcription coactivator activity).

A total of 96 GO terms were enriched using a combination of the two-omics. We selected 20 traits related to those of target to draw a bubble diagram (Figure 3C). Specifically, enriched GO terms possibly related to oxidative stability were positive regulation of biological processes, regulation of response to stimulus, immune response, oxidoreductase activity, acting on the CH-OH group of donors, peroxidase activity, nucleotide biosynthetic process, nucleotide metabolic process, nucleotidyltransferase activity, actin binding, actin filament binding, regulation of actin cytoskeleton organization, lipid binding, phospholipid binding, and Rho GTPase binding.

A heat map was drawn based on the expression levels of 32 differentially expressed proteins (genes) and enriched GO terms (Figure 3D). The main enrichments were metabolic process (*ENSOCUG00000024443*, *ENSOCUG00000024506*, *ACSL6*, *ART3*, *ENSOCUG00000009681*, *ALDH2*, *PRKRA*, *IMPA1*, *GSTM3,* and *PPIB*), structural molecule activity (*LMNB1*, *TUBA1C*, *RPL21*, and *RPL17*), macromolecular complex (*ENO1* and *MYH8*), transferase activity (SHMT1), and cellular process (*SYNPO*, *FSCN1*, and *DBN1*).

### 3.4. Protein Regulatory Network Related to Meat Quality and Oxidative Stability in MBR and CIR

**Figure 4:**
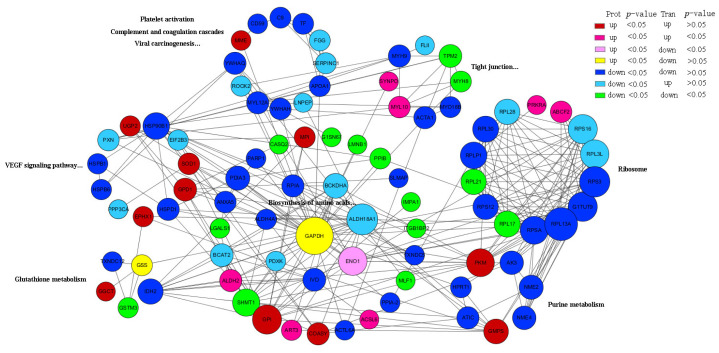
Protein–protein interaction network. Interaction map of key genes between MBR and CIR, where node size corresponds to degree values, and colors indicate expression patterns (proteomic/transcriptomic). Nodes were categorized based on major KEGG and GO pathways, which included glutathione metabolism, VEGF signaling pathway, purine metabolism, amino acid biosynthesis, and other pathways. Red and rose red (*p* < 0.05) represent the upregulation of protein (gene) expression, and dark blue and green (*p* < 0.05) represent the downregulation of protein (gene) expression in MBR than in CIR. Pink and yellow represent protein upregulation and gene downregulation. Light blue represents protein downregulation and gene upregulation.

A total of 327 DEPs at the proteomic level were subjected to bioinformatic analysis. Based on the GO and KEGG enrichment results and a literature review, 102 candidate proteins potentially involved in oxidative stability [26], meat color [27], and flavor [28,29] regulation were selected (Supplementary Table S5). STRING-based protein–protein interaction (PPI) analysis revealed functional interactions between the 88 DEPs (Supplementary Table S5). The interaction network was visualized using Cytoscape 3.10.1 [18], where node size corresponds to degree values and colors indicate expression patterns (proteomic/transcriptomic). The nodes were categorized based on the major KEGG and GO pathways. These proteins predominantly participate in regulatory pathways, including platelet activation, complement and coagulation cascades, viral carcinogenesis, glutathione metabolism, the VEGF signaling pathway, tight junctions, ribosomes, purine metabolism, and amino acid biosynthesis. Twenty-one proteins showed significant differential expression in both transcriptomic and proteomic analyses (represented by magenta and green nodes in Figure 4; *p* < 0.05). In MBR, *MYL10*, *SYNPO*, *ACSL6*, *ART3*, *ALDH2*, *PRKRA*, and *ABCF2* proteins (genes) were upregulated, whereas *MYH8*, *TPM2*, *RPL21*, *RPL17*, *SHMT1*, and others were downregulated, collectively regulating meat flavor. The upregulated SOD1 protein enhanced the antioxidant capacity via peroxidase activity, whereas the downregulated proteins NME2, NME4, HPRT1, AK3, and ATIC, along with the upregulated proteins GMPS and PKM, modulated meat flavor through purine metabolism.

**Figure 5:**
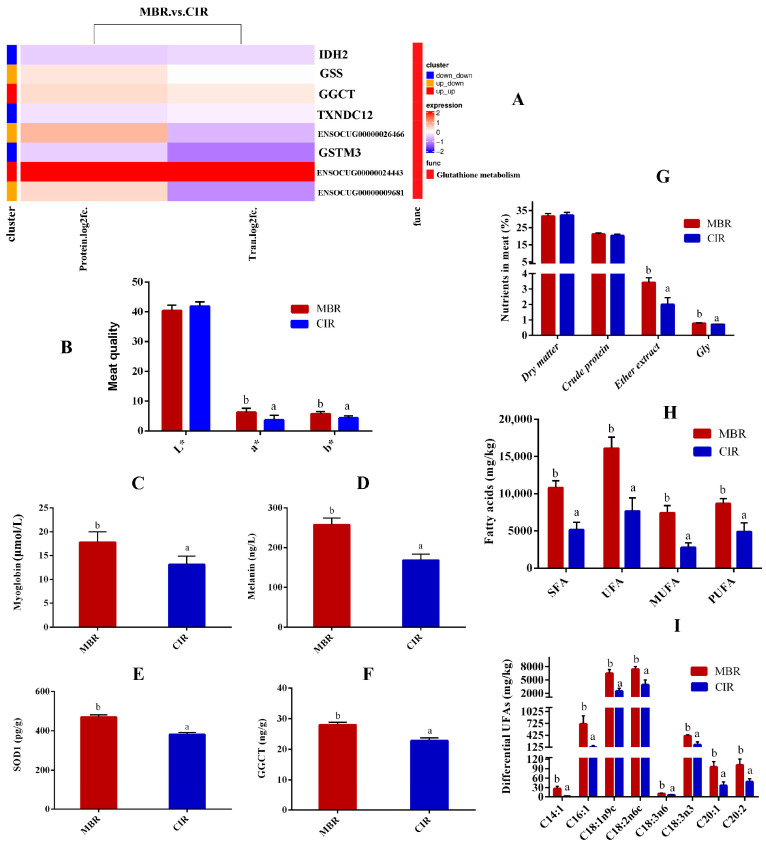
Meat quality-related indicators. (**A**) Glutathione metabolism-related genes KEGG heat map. (**B**) Muscle color index. (**C**) Muscle myoglobin content. (**D**) Muscle melanin content. (**E**) Muscle SOD1 content. (**F**) Muscle GGCT content. (**G**) Muscle nutrient content. (**H**) Different fatty acid contents in muscle. (**I**) Unsaturated fatty acid contents in muscle. The *x*-axis of B represents the meat color index L* (lightness), a* (redness), and b* (yellowness), and the *y*-axis represents the value. The *x*-axis of C-F represents MBR and CIR groups, while the *y*-axis corresponds to myoglobin, melanin, SOD1, and GGCT content, respectively. The *x*-axis of G represents the nutrient composition (dry matter, crude protein, etc.), while the *y*-axis indicates their respective proportions. The *x*-axis of H represents the fatty acid type (SFA, etc.), and the *y*-axis represents its content. The *x*-axis I represents the type of different unsaturated fatty acids, and the *y*-axis represents its content. Different lowercase letters above the bars of peer data indicate significant differences (*p* < 0.05), while no letters indicate no significant differences (*p* > 0.05).

Additionally, the expression of GGCT and GSS proteins was significantly upregulated, whereas that of GSTM3, TXNDC12, and IDH2 was downregulated, which primarily modulated antioxidant traits and meat color through glutathione metabolism. Notably, two novel glutathione metabolism-related proteins, ENSOCUG00000024443 and ENSOCUG00000009681 (annotated as Glutathione S-transferase Mu 2 (GSTM2) and GSTM4, respectively, in the STRING database) exhibited upregulated protein and gene expression (Figure 5A, Supplementary Table S6).

### 3.5. Meat Quality Indicators

Key meat quality parameters were determined based on an integrated multi-omics analysis (Supplementary Table S6). Meat color assessment revealed that the MBR exhibited significantly higher redness (a*), yellowness (b*), melanin, and myoglobin levels compared to those of the CIR (*p* < 0.05; Figure 5B–D). We also measured other physical properties of the muscle (Supplementary Table S7). Consistent with the omics findings, MBR meat showed notably elevated SOD1 and GGCT protein levels (*p* < 0.05; Figure 5E,F), indicating superior antioxidant capacity relative to CIR. Additionally, the MBR demonstrated significantly higher ether extract and glycine (Gly) contents than those of the CIR (*p* < 0.05; Figure 5G).

The fatty acid composition critically influences meat flavor by affecting its aroma, taste, and sensory quality. The MBR exhibited significantly increased levels of saturated fatty acids (SFAs), unsaturated fatty acids (UFAs), monounsaturated fatty acids (MUFAs), and polyunsaturated fatty acids (PUFAs) compared to those of the CIR (*p* < 0.05; Figure 5H). Additionally, comparative analysis of fatty acid profiles revealed that MBR exhibited significantly higher levels of MUFAs including myristoleic acid (C14:1n-5), palmitoleic acid (C16:1n-7), oleic acid (C18:1n-9), eicosenoic acid (C20:1n-9), and eicosadienoic acid (C20:2n-6); and PUFAs including linoleic acid (C18:2n-6, LA), γ-linolenic acid (C18:3n-6, GLA), and α-linolenic acid (C18:3n-3, ALA), compared to those of CIR (*p* < 0.05; Figure 5I).

## 4. Discussion

China’s local breeds, particularly black livestock and poultry, exhibit excellent properties in terms of meat quality and flavor [5,9,30]. In the present study, integrated transcriptomic and proteomic analyses revealed 1175 DEGs and 327 DEPs. Despite the limited number of differentially expressed proteins due to constraints in proteomics technology and the small sample size of omics (*n* = 3), which resulted in fewer significantly enriched KEGG pathways among the top 20 proteomics entries, 17 GO terms showed significant enrichment in the proteomics analysis. Concurrently, pathways that were enriched in both KEGG and GO analyses across the two omics association analyses were integrated. Pathways that primarily regulate meat quality, such as oxidative stability, meat color and flavor, showed enrichment. Furthermore, analysis of meat quality traits confirmed that the MBR, a Chinese indigenous dark-coated breed, exhibits superior meat color and flavor characteristics. By integrating genetic-level insights with phenotypic assessments of meat quality, this pioneering genome-wide investigation systematically elucidates the genetic factors underlying the advantageous traits of MBR, providing a basis for informed conservation strategies. These superior characteristics are primarily manifested in the following aspects.

### 4.1. Antioxidant Capacity

Integrated transcriptomic and proteomic analyses identified key pathways associated with oxidative stability, including peroxidase activity, the HIF-1 signaling pathway, and glutathione metabolism. Li et al. [26] demonstrated through combined proteomic and transcriptomic profiling of pectoral meat that glutathione metabolism and peroxidase activity are critical pathways for enhancing meat antioxidant capacity. Peroxiredoxins, a large conserved family of peroxidases, are the primary cellular defense against oxidative stress in all organisms [31]. This study identified that SOD1 protein, a member of this family, was significantly upregulated in the meat of MBR using proteomics, which was also confirmed by meat ELISA analysis. SOD plays a pivotal role in controlling cellular oxidative stress, which catalyzes the conversion of superoxide radicals into oxygen and hydrogen peroxide [32]. SOD1, also known as Cu/Zn-SOD, has a copper-binding site, which is the catalytic center for pro-oxidant activity, and the zinc-binding site enhances this activity through redox-active metal ions [33]. Studies have indicated its involvement in cellular zinc homeostasis, the regulation of antioxidant gene expression via nuclear transcription factors, and peroxiredoxin-mediated redox signaling [34,35]. In the oxygen-sensing pathway, HIF-1 is activated under low-oxygen conditions, regulating both hypoxia-adaptive gene expression and antioxidant enzyme genes, such as SOD, to enhance cellular antioxidant defenses [36].

Additionally, glutathione metabolism-mediated signaling is involved in the regulation of reactive oxygen species (ROS) in living cells in dynamic and challenging environments [37]. Proteomic analysis in this study revealed upregulated GGCT levels in MBR meat, which were further confirmed by ELISA. GGCT is a key enzyme in maintaining glutathione (GSH) homeostasis and is one of the primary enzymes involved in glutathione catabolism [38]. He et al. [39] reported that *GGCT* deficiency in mice led to splenomegaly and progressive anemia phenotypes that were attributed to increased ROS levels in red blood cells and increased oxidative damage caused by *GGCT* deletion. Moreover, GGCT deficiency resulted in reduced levels of both GSH and its biosynthetic precursor cysteine, indicating that GGCT is crucial in the erythrocyte redox balance by regulating glutathione metabolism. Additionally, we identified two novel genes associated with GSH metabolism pathways, *ENSOCUG00000024443* and *ENSOCUG00000009681* (annotated as *GSTM2* and *GSTM4*, respectively, in the STRING database), which exhibited upregulated protein expression in MBR. GSTM2, GSTM3, and GSTM4, which are members of the glutathione S-transferase (GST) family, are essential for detoxifying chemical substrates (toxins and carcinogens) and managing oxidative stress [40,41]. GSTM2, a phase II metabolic enzyme, conjugates glutathione to electrophilic compounds and is vital for detoxifying harmful chemicals [42]. GSTM2 also contributes to cellular antioxidant defense by protecting against oxidative damage and cell death [43]. GSTM4 is another critical detoxification enzyme that catalyzes the conjugation of GSH to diverse electrophilic substrates and modulates cellular redox responses [44]. Additionally, we enriched the melanogenesis signaling pathway, and correspondingly, the melanin content in the MBR meat was significantly higher than that in the CIR meat. Melanin exhibits exceptional natural antioxidant properties [45]. This also confirmed the superior antioxidant capacity of the MBR meat. Based on these findings, MBR meat exhibits superior antioxidant capacity than that of CIR meat, which is mediated through pathways involving peroxidase activity and glutathione metabolism. Notably, SOD1 and GGCT may serve as key regulatory factors in this process.

### 4.2. Meat Color

Meat color is a primary indicator of meat quality and is a crucial commercial sensory indicator. The b* value of the meat indicates its freshness [46]. Color is a vital standard for evaluating the freshness, quality, and acceptability of red meat. The b* value of MBR was higher than that of CIR, indicating that the freshness of MBR meat immediately post-slaughter exceeds that of CIR meat, which is consistent with the findings by Liu et al. [47]. The L* value was negatively correlated with melanin content. Zi et al. [48] demonstrated that the melanin content was significantly higher in black-feathered chickens than in white-feathered chickens, whereas the latter exhibited significantly high L* values. ELISA results further revealed that the MBR showed higher melanin content and lower L* values than those of the CIR, which is consistent with these findings. The a* value of meat depends on its myoglobin content, which is a key pigment and the most notable factor in maintaining red meat coloration [49,50]. In this study, the myoglobin content in the MBR was higher than that in the CIR. Combined with the superior meat a* values observed in the MBR group, these results demonstrate that the MBR exhibits superior meat color characteristics than those of the CIR. This corroborates previous studies that MBR meat exhibits a more vibrant red coloration than that of New Zealand meat rabbits [51], indicating the unique advantages of indigenous black breeds in terms of meat color attributes. Additionally, Laiwu black rabbits exhibit superior meat color quality than that of white rabbits, and increased consumer preference ratings [5]. These findings collectively highlight the distinct advantages of indigenous black breeds in terms of meat color attributes.

Our multi-omics analyses revealed enrichment of metabolism-cytochrome P450, glutathione metabolism signaling pathways, and melanogenesis, suggesting their potential involvement in the regulation of meat color. Seo et al. [27] demonstrated through RNA-seq analysis that cytochrome P450 and glutathione peroxidase 5 (GPX5) were dose-dependently upregulated in porcine cell lines cultured with heme supplementation. Heme, a critical biomolecule in meat tissue that is predominantly localized in myoglobin, is integral in determining meat color (vivid redness) and flavor development [52]. These findings provide preliminary evidence for the mechanistic convergence of these pathways in meat color regulation. The glutathione system is further implicated in pheomelanogenesis, where GSH thiol groups react with dopaquinone to form cysteinyl-dopa conjugates, ultimately yielding sulfur-containing pheomelanin pigments [53]. Pheomelanin regulates feather coloration in poultry. Furthermore, it directly participates in the generation of ROS within melanocyte mitochondria, thereby affecting cellular oxidative stress and systemic damage in organisms [54]. Wang et al. [55] reported that dietary supplementation with 0.3% chestnut tannin in lambs enhanced their antioxidant capacity, mitigated oxidative stress, and improved meat color stability and tenderness. In this study, we identified key proteins/genes (*GSTM3*, *ENSOCUG00000024443* and *ENSOCUG00000009681*) that were co-enriched in both glutathione metabolism and cytochrome P450 pathways. Notably, the latter two genes showed upregulated expression in the MBR than in the CIR. We speculate that these molecules may alleviate oxidative stress through the coordinated regulation of antioxidant and detoxification pathways, thereby contributing to the superior meat color characteristics observed in MBR.

### 4.3. Meat Flavor

Meat flavor is a critical determinant of meat consumption, and lipids are the primary precursors of flavor compounds. The composition of fatty and amino acids notably affects meat flavor, juiciness, and nutritional value [28,29]. In this study, pathways, including PPAR signaling, fatty acid biosynthesis, fat digestion and absorption, tight junctions, amino acid biosynthesis, amino sugar and nucleotide sugar metabolism, and purine metabolism, were associated with flavor regulation. Upregulation of the PPAR signaling pathway is accompanied by increased lipid accumulation [56]. Through transcriptomic analysis, Wang et al. [57] reported that PPAR signaling is a key lipid metabolism pathway that potentially improves beef quality by influencing intramuscular fat content in cattle. Fatty acid biosynthesis, digestion, and absorption are closely related to meat tenderness, juiciness, and flavor formation [58]. Association analysis revealed that the *Acyl-CoA synthetase long chain family member 6* (*ACSL6*) gene/protein was significantly upregulated. The ACSL6 enzyme encoded by this gene is a core hub for the regulation of fatty acid metabolism and is involved in lipid synthesis, energy metabolism, and cell signal transduction [59]. Shi et al. [60] observed that the expression of ACSL6 was positively correlated with the content of docosahexaenoic acid (DHA), an ω-3 PUFA, in meat, affecting the meat flavor of *Oncorhynchus mykiss*. Additionally, upregulated *MYL10* (gene/protein) was identified in the tight junction pathway of MBR. Chen et al. [61] reported a positive correlation between *MYL10* and intramuscular fatty acid levels, indicating its potential role in regulating fatty acid content and flavor development. We further determined the fatty acid content of the meat and observed that the MBR meat contained significantly increased levels of eight UFAs: C14:1, C16:1, C18:1n9c (oleic acid), C18:2n6c (linoleic acid), C18:3n6, C18:3n3, C20:1, and C20:2. UFAs oxidize more readily than SFAs, with high concentrations enhancing the flavor intensity. Monounsaturated fatty acids undergo oxidation to generate precursors (aldehydes and furans) that contribute to aroma formation [62]. Notably, C18:1n9c and linoleic acid C18:2n6c oxidatively degrade into aldehyde-based flavor compounds, forming meaty aromas [63]. The total oleic and linoleic acid content serves as a key indicator of fatty acid-driven flavor impact. Yu et al. [64] identified oleic acid [C18:1(n-9)] and linoleic acid [C18:2(n-6)] as the dominant fatty acids influencing beef flavor. Unsaturated fatty acids such as oleic, linoleic, and linolenic acids further modulate early-stage Maillard reactions (“glucose-glutathione” pathways) and the formation of meaty flavor compounds [65]. Therefore, the increased levels of unsaturated fatty acids, such as C18:1n9c, C18:2n6c, and C18:3n6, in MBR meat are a key factor contributing to its distinctive flavor profile.

Glycine, a conditionally restricted nonessential amino acid, exerts important regulatory effects on lipid metabolism [66]. Recent studies have highlighted glycine metabolism as a key marker of meat flavor quality in aged ducks [67]. The significantly higher glycine content in the MBR than in the CIR indicates superior flavor characteristics of the MBR. Picard et al. [68] demonstrated that *ENO1* is associated with meat tenderness. Previous studies have shown that the tenderness of MBR meat is better than that of CIR meat [5]. In addition, multi-omics correlation analysis revealed upregulated ENO1 protein expression in the amino acid biosynthesis pathway in MBR, suggesting that ENO1 may enhance meat tenderness in MBR, thereby improving textural quality. These findings demonstrate that fatty and amino acid compositions considerably influence meat flavor characteristics.

Additionally, the purine metabolism pathway enriched in our multi-omics data may be involved in flavor regulation. Tansutaphanit et al. [69] demonstrated that purines directly influence fatty acid composition by modifying fatty acid metabolism-related genes in the liver of rainbow trout, highlighting the close interplay between purines and fatty acid metabolism. The de novo purine biosynthesis pathway in prokaryotes comprises 10 enzymatic steps: conversion of phosphoribosyl pyrophosphate (PRPP) to inosine monophosphate (IMP), which is directly synthesized into adenosine monophosphate (AMP) or guanosine monophosphate (GMP) [70]. AMP undergoes catabolic conversion to hypoxanthine, a critical indicator of meat degradation. High IMP levels (associated with umami) and relatively low hypoxanthine content (associated with bitterness) are desirable for flavor [71]. We compared the hypoxanthine content in the two rabbit meat breeds and found that the hypoxanthine content in MBR was significantly lower than that in CIR (Supplementary Table S8), indicating that MBR likely has superior umami flavor characteristics. This study identified the key proteins, NME2 and NME4, in this pathway that may be associated with meat flavor characteristics, both showing downregulated expression in MBR. Iuso et al. [72] demonstrated that NME2 negatively regulated fatty acid accumulation. Xie et al. [73] revealed that NME4 plays a central role in mitochondrial lipid metabolism. Cai et al. [74] enhanced the deposition of flavor compounds in meat by supplementing the meat with substrates for nucleotide synthesis to activate the purine metabolic pathway. These findings suggest that purine metabolism regulates meat quality and flavor differences between MBR and CIR breeds, with NME2 and NME4 proteins playing key roles in these processes. These proteins may serve as potential breeding targets to improve the quality of rabbit meat.

## 5. Conclusions

The integrated transcriptomic and proteomic analysis, combined with phenotypic analysis, conclusively demonstrated that the indigenous MBR breed exhibits superiority in part of meat quality indicators. Transcriptomic and proteomic analyses identified enrichment of antioxidant pathways (glutathione metabolism, peroxidase activity, and HIF-1 signaling) within the MBR. The key upregulated proteins, SOD1 and GGCT, validated by ELISA, enhanced the intrinsic antioxidant capacity. *GSTM3, ENSOCUG00000024443* and *ENSOCUG00000009681*, which act at the interface of glutathione metabolism and cytochrome P450 pathways, likely improve meat color stability by mitigating oxidative stress. Phenotypic analysis confirmed significantly superior color attributes in the MBR, including increased redness (a*), yellowness (b*), and melanin and myoglobin contents. Additionally, MBR meat exhibited significantly increased levels of unsaturated fatty acids (UFAs) and glycine, key flavor compounds. Proteins involved in fatty acid (ACSL6), purine (NME2 and NME4), tight junction (MYL10), and amino acid metabolism (ENO1) may be involved in regulating these desirable flavor precursors. Collectively, these findings highlight the intrinsic value of MBR as a genetic resource with desirable meat quality traits, particularly enhanced antioxidant properties, color stability, and flavor profiles. This comprehensive assessment provides a robust scientific foundation for optimizing the conservation and utilization strategies for this valuable indigenous genetic resource. Currently, the advantages of MBR have only been confirmed through omics studies, yet the regulatory mechanisms underlying these beneficial traits remain insufficiently explored. In the future, we will continue to conduct in-depth research on the meat quality and antioxidant mechanisms of the MBR so as to provide guidance for the development and utilization of this indigenous breed.

## Data Availability

Data will be made available upon reasonable request. The raw sequence data reported in this paper have been deposited in the Genome Sequence Archive and OMIX at the National Genomics Data Center (Nucleic Acids Res 2022), China National Center for Bioinformation/Beijing Institute of Genomics, and Chinese Academy of Sciences, which that are publicly accessible at https://ngdc.cncb.ac.cn/gsa (accessed on 23 June 2025). Transcriptome data were obtained from GSA: CRA027355, https://ngdc.cncb.ac.cn/gsa/s/69H0kFt3 (accessed on 23 June 2025). Proteomics data were obtained from OMIX010830, https://ngdc.cncb.ac.cn/omix/preview/BJM7qG7M (accessed on 2 July 2025).

## Supplementary Materials

The following supporting information can be downloaded at: https://www.mdpi.com/article/10.3390/ani15243616/s1, Table S1: Comparison and nutrient levels of diets (air-dry basis, %); Table S2: Data of differentially expressed genes (DEGs) and proteins (DEPs) in MBR vs. CIR; Table S3: Data of KEGG enrichment analysis; Table S4: Data of GO enrichment analysis; Table S5: Data of protein–protein interaction network; Table S6: Data of meat quality-related indicators; Table S7: Comparison of muscle physical properties in MBR and CIR; Table S8: Comparison of Nucleotide Contents in MBR and CIR meat.

## Abbreviations

The following abbreviations are used in this manuscript:

MBR	Minxinan black rabbit
CIR	Hyla rabbit
DEGs	differentially expressed genes
DEPs	differentially expressed proteins
DAMs	differentially accumulated metabolites
SOD1	superoxide dismutase 1
GGCT	gamma (γ)-glutamylcyclotranserase
LTL	longissimus thoracis et lumborum
FAME	fatty acid methyl ester
GC–MS	Gas chromatography–mass spectrometry
TMT	Tandem Mass Tag
ELISA	Enzyme-linked immunosorbent assay
PPI	protein–protein interaction
SFAs	saturated fatty acids
UFAs	unsaturated fatty acids
MUFAs	monounsaturated fatty acids
PUFAs	polyunsaturated fatty acids
ROS	reactive oxygen species
