# Supplementary material for: Comparative Analysis of Meat Quality in Minxinan Black Rabbit and Hyla Rabbit Using Integrated Transcriptomics and Proteomics
Source: Animals (Basel). 2025 Dec 16;15(24):3616. doi: 10.3390/ani15243616 (PMC12730085; doi:10.3390/ani15243616)
Supplement: Supplementary file 1 [file animals-15-03616-s001.zip › Supplementary Table S1 Comparison and nutrient levels of diets; S7 Comparison of muscle physical properties in MBR and CIR; S8 Comparison of Nucleotide Contents in MBR and CIR meat .docx]

**Supplementary Materials**

The basic diet composition and nutritional level of Minxinan black rabbit (MBR) and Hyla rabbit (CIR) (Table S1).

**Supplementary Table S1.** Comparison and nutrient levels of diets (air-dry basis, %)

| **Ingredients** | **Content** | **Nutrient levels** ^2^ | **Content** |
| --- | --- | --- | --- |
| Corn | 25.0 | Digestible energy /(MJ kg-1) | 9.90 |
| Soybean meal | 17.0 | Crude protein | 16.26 |
| Wheat bran | 11.0 | Neutral-detergent fibre | 31.27 |
| Wheat middling | 10.0 | Acid detergent fibre | 20.39 |
| Alfalfa meal | 8.0 | Acid detergent lignin | 6.26 |
| Peanut vine | 8.0 | Crude fat | 2.55 |
| Peanut shell | 19.0 | Calcium | 0.84 |
| Calcium hydrogen phosphate | 0.5 | Total phosphorus | 0.44 |
| Calcium carbonate | 0.5 | Lysine | 0.79 |
| Premix ^1^ | 1.0 | Methionine and cysteine | 0.56 |
| Total | 100.0 |  |  |

^1^ The premix provided the following per-kilogram diet: vitamin A: 10 000 IU, vitamin D3: 1500 IU, vitamin E: 50 mg, vitamin K3: 3.0 mg, thiamine: 5.0 mg, riboflavin: 10 mg, pantothenic acid: 20 mg, nicotinic acid: 50 mg, Fe: 100 mg, Zn: 30 mg, Cu: 20 mg, Mn: 30 mg, choline: 400 mg, NaCl: 5.0 g, lysine: 1.0 g, methionine: 1.0 g. The rest is a miscellaneous meal carrier complement.

^2^ Digestible energy was a calculated value, while the others were measured values.

The pH_24h_, shear force and water loss ratio of the MBR muscle were all significantly higher than those of the CIR muscle (*p*<0.05, Table S7).

**Supplementary Table S7.** Comparison of muscle physical properties in MBR and CIR

| **Items** | **MBR^1^** | **CIR^1^** | ***P*-value** |
| --- | --- | --- | --- |
| pH_45min_ | 6.68±0.08 | 6.69±0.09 | 0.735 |
| pH_24h_ * | 5.88±0.11 ^b^ | 5.72±0.09 ^a^ | 0.001 |
| Shear force /kg.f * | 29.01±3.56 ^b^ | 24.25±2.28 ^a^ | 0.002 |
| Drip loss ratio /% | 1.19±0.70 | 0.97±0.31 | 0.391 |
| Water loss ratio /% * | 45.38±2.17 ^b^ | 41.53±3.15 ^a^ | 0.005 |
| Cooked meat ratio/% | 64.06±1.62 | 62.72±2.15 | 0.132 |

* Different lowercase letters on the shoulder labels of peer data indicate significant differences (*p<0.05*), while no letters indicate no significant differences (*p>0.05*). Same below.

^1^ The MBR represents Minxinan black rabbit, and the CIR represents Hyla rabbit. Same below.

Comparison of nucleotides in meat was performed using Agilent 1200 liquid chromatography system (Agilent Technologies Inc., Santa Clara, CA, USA), and the hypoxanthine content in the MBR was significantly lower than that in the CIR (*p<*0.05, Table S8).

**Supplementary Table S8.** Comparison of Nucleotide Contents in MBR and CIR meat.

| **Items/(mg/kg)** | **MBR** | **CIR** | ***P*-value** |
| --- | --- | --- | --- |
| Cytosine | 4.73±0.42 | 4.89±0.94 | 0.773 |
| Uracil | 28.51±1.98 | 33.78±6.66 | 0.180 |
| Guanine | 44.80±2.77 | 41.65±1.57 | 0.095 |
| Adenine | 2268.90±110.21 | 2102.78±543.97 | 0.571 |
| Hypoxanthine | 152.75±21.06 ^a^ | 184.46±14.31 ^b^ | 0.047 |
